# Medical Men and Midwives

**Published:** 1909-11-06

**Authors:** 


### MEDICAL MEN AND MIDWIVES.

The recommendations of the Departmental Committee appointed by the Lord President of the Privy Council to consider the working of the Midwives' Act, 1902, are as follows:—

"A secure expectation of payment should be given to a medical practitioner summoned on the advice of a midwife in a case of emergency. The poor law authority should be responsible for the payment, and should be empowered to charge the fee paid as 'relief on loan' to the patient. The scale of fees should be fixed by order of the Local Government Board on a systematic basis, having due regard to local conditions. In emergency cases the system of a uniform fee, which should cover any ordinary after-attendance, is recommended for adoption, with the addition, perhaps, in scattered rural areas, of some allowance for mileage. The medical practitioner, in applying to the public authority, should be required to state that he has been unable to obtain payment of his fee from the patient or her representatives. A list of practitioners willing to respond to a midwife's call should be prepared and circulated in every area." The Midwives Committee of the London and Counties Medical Protection Society recommends, and the Council of the Society has approved the recommendation:—"That, having regard to the proposed payment by Boards of Guardians of the doctors called in to assist midwives, the London and Counties Medical Protection Society recommends the medical men in each Union to meet and confer on the subject, and to agree as to what fee, or fees, they will accept, and then combine to insist on such fee or fees being paid (or guaranteed) by the Guardians, and paid on the statement of the medical man that he is unable to obtain payment of his fee from the patient or her representatives."

The subject is an important one, and should have the consideration of every general practitioner.

